# Wedge resection versus lobectomy in T1 lung cancer patients: a propensity matched analysis

**DOI:** 10.1186/s13019-023-02303-4

**Published:** 2023-08-24

**Authors:** Robert J Moon, Rebecca Taylor, Pika Miklavc, Syed B Mehdi, Stuart W Grant, Mohamad Nidal Bittar

**Affiliations:** 1 Department of Cardiothoracic Surgery, Lancashire Cardiac Centre, Blackpool Victoria Hospital, Blackpool, UK; 2 School of Science, Engineering and Environment, University of Salford, Manchester, UK; 3 Department of Respiratory Medicine, Lancashire Teaching Hospitals NHS Foundation Trust, Preston, UK

**Keywords:** Lung, Cancer, Resection, Lobectomy, Wedge, Survival

## Abstract

**Objectives:**

Performing wedge resection rather than lobectomy for primary lung cancer remains controversial. Recent studies demonstrate no survival advantage for non-anatomical resection compared to lobectomy in patients with early-stage lung cancer. The objective of this study was to investigate whether in patients with T1 tumours, non-anatomical wedge resection is associated with equivalent survival to lobectomy.

**Methods:**

This was a retrospective cohort study of patients who underwent lung resection at the Lancashire Cardiac Centre between April 2005 and April 2018. Patients were subjected to multidisciplinary team discussion. The extent of resection was decided by the team based on British Thoracic Society guidelines. The primary outcome was overall survival. Propensity matching of patients with T1 tumours was also performed to determine whether differences in survival rates exist in a subset of these patients with balanced pre-operative characteristics.

**Results:**

There were 187 patients who underwent non-anatomical wedge resection and 431 patients who underwent lobectomy. Cox modelling demonstrated no survival difference between groups for the first 1.6 years then a risk of death 3-fold higher for wedge resection group after 1.6 years (HR 3.14, CI 1.98–4.79). Propensity matching yielded 152 pairs for which 5-year survival was 66.2% for the lobectomy group and 38.5% for the non-anatomical wedge group (SMD = 0.58, p = 0.003).

**Conclusions:**

Non-anatomical wedge resection was associated with significantly reduced 5-year survival compared to lobectomy in matched patients. Lobectomy should remain the standard of care for patients with early-stage lung cancer who are fit enough to undergo surgical resection.

## Significance

**What is already known on this topic:**

There is conflicting data regarding the use of non-anatomical resection in early-stage lung cancer in terms of long-term outcomes. This study was done to determine survival outcomes of non-anatomical resection versus lobectomy.

**What this study adds:**

Lobectomy should remain the standard of care for patients with early-stage lung cancer who are fit enough to undergo surgical resection.

**How this study might affect research, practice or policy:**

Surgeons should offer lobectomy in all patients except in patients who cannot tolerate it due to poor cardiopulmonary reserve.

## Introduction

Lung cancer has one of the poorest 5-year survival rates amongst all cancers. This has not improved much in the last three decades and remains around 15% [1]. Surgery can often be curative however the majority of patients present at a late stage where surgery has no role to play. Early detection has been studied over the last two decades and the need for lung cancer screening has been piloted in the UK [2]. Surgery is offered to stage I and II lung cancer patients and lobectomy has been the gold standard surgical procedure in the management of early stage lung cancer based upon the only randomised control trial comparing the two procedures in 1995 [3]. Non-anatomical wedge resection was found to be associated with a higher rate of recurrence at 5 years [3]. Historically, non-anatomical wedge resection has been offered based on clinical judgment taking into consideration the patient’s performance status, lung function and comorbidities. The last decade witnessed massive adoption of video assisted thoracoscopic (VATS) procedures, initiating revolution in the surgical management of lung cancer with some units in the UK achieving 90% of total resections by VATS surgery [4].

Recent published data showed no survival advantages for non-anatomical wedge versus lobectomy in octogenarian patients in early stage lung cancer [5, 6]. As a result of this emerging evidence and smaller tumours being picked up by screening, the choice between sub-lobar and anatomical resection is very much influenced by the surgeon and institutions experience. Segmentectomy is considered to be superior to wedge resection due to improved lymph node clearance and larger tumour margin which leads to higher survival rates [7]. Data from the National Lung Cancer Audit classifies segmentectomy and wedge resection under a ‘sub-lobar’ category which does not distinguish them as separate entities [8], although many thoracic surgeons would not class them as oncologic equivalent procedures [9].

European guidelines recommend that anatomical resection should be performed over wedge resection and that lobectomy should still be the standard of care in patients with tumour size of 2 cm or greater [10]. American guidelines suggest that parenchymal sparing procedures may infer benefit to those who are aged over 75 years [11]. In contrast, British guidelines advise that age over 80 alone should not be a contraindication to lobectomy [12]. It is worth noting that a number of these guidelines have not been updated for some time and surgical techniques, as well as adjuvant therapy have vastly improved. In the UK, lobectomy is the mainstay of treatment, accounting for around 77% of surgeries, followed by wedge resection at around 19% [8].

We have retrospectively analysed our centre’s data and compared outcomes between the two approaches to investigate whether in patients with T1 tumours, non-anatomical wedge resection is associated with equivalent long-term survival.

## Patients and methods

This is a retrospective cohort study of all patients with T1 tumours who underwent lung resection (excluding pneumonectomy) at the Lancashire Cardiac Centre, Blackpool Victoria Hospital between April 2005 and April 2018. Data was prospectively collected by an audit officer in the department and uploaded to the cardiothoracic Patient Advocate Tracking System (PATS) for audit purposes [13]. This database holds national data in the UK for patients undergoing thoracic surgery including those at Blackpool Victoria Hospital. Long term survival was obtained from the Personal Demographics Service (PDS) of the National Health Service (NHS) patients, and data was extracted in 2018. Follow up of patients was until February 2019. The study was approved by the Blackpool Teaching Hospitals NHS Foundation Trust Research and Development committee; taking the form of a service evaluation no ethical review was required.

Segmentectomy is seldom performed at our centre. As a result, for the purposes of this study the term ‘wedge’ refers to a non-anatomical sub-lobar resection and excludes segmentectomy. All sub-types of histology were included. Tumour staging was done using the 7th edition of TNM classification as data was taken over a long time frame [14]. Our department adopted the TNM 8th Edition in 2018 [15].

### Statistical analysis

Differences between relevant pre- and post-operative characteristics are explored and described as “frequency (%)” for categorical variables and “median [Q1, Q3]” for continuous variables. The magnitude of difference is quantified using Standardised Mean Difference (SMD) and differences tested for significance using Fisher’s Exact tests or Mann-Whitney U tests for categorical and continuous variables respectively, with a SMD < 0.1 deemed good balance and p < 0.05 deemed statistically significant. All continuous variables were found to be non-normally distributed (Kolmogorov-Smirnov test p < 0.05).

A Cox proportional hazards model was fit to the data set of all patients with T1 tumours. All pre-operative characteristics deemed likely to affect either the choice of procedure or survival were included in the initial model and then retained or dropped using a process of backward selection. Exploratory Kaplan-Meier analyses showed a potential deviation from the assumption of proportional hazards, this was verified using a test of the independence of Schoenfeld residuals for the treatment covariate with time. Thus, the Cox model was refitted with a time-varying coefficient of treatment group; the placement of this cut off is tested over a continuum of time thresholds and the model with the lowest AIC selected.

### Propensity matching

In recognising the presence of indication bias, in that the surgical procedure decision may depend on certain patient characteristics, a propensity matched set of T1 stage tumour patients was created, matching on the variables most influential in the choice of procedure; namely age (under or at least 70 years old), sex, predicted FEV1, and performance status. Due to missing FEV1 and performance status data, the pool of potential patients for matching was reduced from 618 to 536. Matching was performed using logistic propensity scores and nearest-neighbour matching in a 1–1 ratio, using a caliper of width 0.2 times the standard deviation of the propensity scores. The “MatchIt” package in R was used [16]. The matching process led to a set of 152 pairs being matched across those variables. Post-matching balance on the covariates used is good with SMD across surgical groups for these covariates greatly reduced to close to or below the target SMD of 0.1. This matched set describes a cohort for whom wedge resection and lobectomy are both viable choices and who are sufficiently balanced to be expected to have similar outcomes. Several variables in Table 1 do still display unacceptable balance after matching.

**Table 1:** Characteristics of patients with t1 tumours, before and after propensity matching. SMD – standardised mean difference

					Before matching			After matching *(missing cases are excluded from variables used in matching procedure)*			
		Characteristic *(Number missing for Lobectomy and Wedge groups respectively, if not zero, before matching)*		Lobectomy (n = 431)	Wedge resection (n = 187)	SMD	p	Lobectomy (n = 152)	Wedge resection (n = 152)	SMD	p
PRE-OPERATIVE		Age, years	Age in years Over 70	68 [62, 74] 189 (43.9%)	72 [68, 78] 123 (65.8%)	0.54 0.45	< 0.001 < 0.001	72 [67,76] 100 (65.8%)	72 [68,77] 97 (63.8%)	0.15 0.04	0.21 0.72
		Sex	Female	226 (52.4%)	106 (56.7%)	0.09	0.38	85 (55.9%)	83 (54.6%)	0.026	0.91
		BMI *(8, 1)*	Overall	26 [23, 29]	27 [23, 30]	0.05	0.54	26.5 [23, 30]	26.5 [23, 30]	0.10	0.38
			Underweight	7 (1.6%)	7 (3.7%)	0.15	0.34	4 (2.6%)	7 (4.6%)	0.18	0.51
			Normal	156 (36.2%)	66 (35.3%)			50 (32.9%)	50 (32.9%)		
			Overweight	169 (39.2%)	68 (36.4%)			64 (42.1%)	54 (35.5%)		
			Obese	91 (21.1%)	45 (24.1%)			33 (21.7%)	40 (26.3%)		
		Smoking status *(1, 0)*	Never	48 (11.1%)	16 (8.6%)	0.09	0.41	15 (9.9%)	14 (9.2%)	0.02	> 0.99
			Current/ex	382 (88.6%)	171 (91.4%)			137 (90.1%)	138 (90.8%)		
		Urea, mmol/L *(8, 0)*		5.5 [4.4, 6.6]	6.1 [4.6, 7.5]	0.10	0.28	5.7 [4.7, 6.7]	6.2 [4.6,7.4]	0.01	0.91
		Creatinine, µmol/L *(7, 0)*		80 [70, 98]	83 [66, 97]	0.10	0.25	80 [69, 95]	83 [66, 97]	0.08	0.46
		Haemoglobin, g/dl *(7, 0)*		13.8 [12.8, 14.7]	13.6 [12.3, 14.6]	0.05	0.61	13.7 [12.6, 14.5]	13.6 [12.3, 14.5]	0.11	0.33
		Diabetes status *(1. 0)*	No	379 (87.9%)	159 (85.0%)	0.09	0.35	127 (83.6%)	130 (85.5%)	0.06	0.75
			Yes (any)	51 (11.9%)	28 (15.0%)			25 (16.4%)	22 (14.5%)		
		Ischaemic heart disease *(7, 5)*	Yes	72 (16.7%)	43 (23.0%)	0.17	0.07	36 (23.7%)	34 (22.4%)	0.03	0.92
		Cardiac failure *(6, 5)*	Yes	6 (1.4%)	8 (4.3%)	0.18	0.051	0	7 (4.6%)	0.31	0.022
		Previous stroke (*2, 2)*		39 (9.0%)	16 (8.6%)	0.02	0.98	22 (14.5%)	10 (6.6%)	0.26	0.042
		ECOG Performance Status *(7, 3)*	Fully active	265 (61.5%)	86 (46.0%)	0.35	0.001	82 (53.9%)	75 (49.3%)	0.12	0.79
			Mobile > 50%	27 (6.3%)	25 (13.4%)			12 (7.9%)	16 (10.5%)		
			Light work	124 (28.8%)	67 (35.8%)			54 (35.5%)	56 (36.8%)		
			Limited/immobile	8 (1.9%)	6 (3.2%)			4 (2.6%)	5 (3.3%)		
		ASA grade *(42, 25)*	Normal healthy	175 (40.6%)	38 (20.3%)	0.51	< 0.001	59 (38.8%)	37 (24.3%)	0.41	-
			Mild disease	161 (37.4%)	80 (42.8%)			58 (38.2%)	65 (42.8%)		
			Severe disease	52 (12.1%)	43 (23.0%)			16 (10.5%)	32 (21.1%)		
			Incapacitating/life threatening	1 (0.2%)	1 (0.5%)			0	0		
		FEV1% predicted *(39, 25)*		86 [70, 98]	75 [59, 90]	0.36	< 0.001	77 [64, 93]	76.5 [60, 92]	0.01	0.91
		FVC % predicted *(70, 37)*		100 [86, 112]	98 [85, 115]	0.09	0.26	96 [83, 110]	98 [85, 113]	0.11	0.37
		Operative priority	Elective	357 (82.8%)	155 (82.9%)	0.002	> 0.99	130 (85.5%)	126 (82.9%)	0.07	0.64
			Emergency/urgent	74 (17.2%)	32 (17.1%)			22 (14.5%)	26 (17.1%)		
POST OPERATIVE		ITU length of stay *(54, 18)*		1 [1, 2]	1 [1, 2]	0.001	0.53	1 [1, 2] max = 76 days	1 [1, 2] max = 62 days	0.07	0.26
		Total length of stay		7 [5, 10] max 76 days	5 [4, 8] max 48 days	0.25	0.004	7 [6, 10] max 76 days	5.5 [4, 8] max 48 days	0.35	0.002
		Post op complications	Yes	72 (17.4%)	22 (11.8%)	0.14	0.15	31 (20.4%)	17 (11.2%)	0.26	0.041
		Ventilation *(1, 0)*	Yes	13 (3.0%)	7 (3.7%)	0.04	0.83	6 (3.9%)	6 (3.9%)	< 0.001	> 0.99
		Air leak	Yes	47 (10.9%)	15 (8.0%)	0.10	0.34	20 (13.2%)	11 (7.2%)	0.20	0.13
		Pleural effusion	Yes	6 (1.4%)	2 (1.1%)	0.03	> 0.99	2 (1.3%)	2 (1.3%)	< 0.001	> 0.99
		Infection	Yes	41 (9.5%)	16 (8.6%)	0.03	0.82	21 (13.8%)	13 (8.6%)	0.17	0.20
		Return to theatre	Yes	9 (2.1%)	1 (0.5%)	0.14	0.29	2 (1.3%)	1 (0.7%)	0.07	> 0.99
		Histology *(1, 0)*	Adenocarcinoma	247 (57.3%)	101 (54.0%)	0.34	0.04	88 (57.9%)	81 (53.3%)	0.32	-
			Squamous cell	90 (20.9%)	57 (30.5%)			36 (23.7%)	46 (30.3%)		
			Non-small-cell	18 (4.2%)	3 (1.6%)			5 (3.3%)	3 (2.0%)		
			Brochiolo-alveolar	14 (3.2%)	1 (0.5%)			5 (3.3%)	1 (0.7%)		
			Small cell	3 (0.7%)	3 (1.6%)			0	3 (2.0%)		
			Undifferentiated	1 (0.2%)	0			0	0 (0.4%)		
			Other	1 (0.2%)	0			17 (11.2%)	18 (11.8%)		
		Post-operative T-stage (*32, 18)*	T1	285 (66.1%)	131 (70.1%)	0.21	0.12	95 (62.5%)	108 (71.1%)	0.32	0.036
			T2	98 (22.7%)	29 (15.5%)			40 (26.3%)	22 (14.5%)		
			T3-4	15 (3.5%)	9 (4.8%)			6 (3.9%)	9 (5.9%)		
		Post-operative N-stage *(38, 41)*	N0	318 (73.8%)	115 (61.5%)	0.05	0.66	115 (75.7%)	91 (59.9%)	0.16	0.28
			N1-2-X	75 (17.4%)	31 (16.6%)			23 (15.1%)	27 (17.8%)		
		Post-operative M-stage *(83, 47)*	M0	253 (58.7%)	88 (47.1%)	0.21	0.10	85 (55.9%)	70 (46.1%)	0.12	0.68
			M1	5 (1.2%)	3 (1.6%)			2 (1.3%)	2 (1.3%)		
			MX	90 (20.9%)	49 (26.2%)			37 (24.3%)	39 (25.7%)		
SURVIVAL		In-hospital mortality		6 (1.4%)	0	0.17	0.19	2 (1.3%)	0	0.16	0.50
		Survival	30 days	426/431 98.8%	187/187 100%	0.15	0.33	151/152 99.3%	152/152 100%	0.12	> 0.99
			1 year	386/411 93.9%	152/171 88.9%	0.18	0.057	133/145 91.7%	128/140 91.4%	0.01	0.96
			5 years	133/207 64.3%	21/58 36.2%	0.58	< 0.001	47/71 66.2%	20/52 38.5%	0.58	0.003
			10 years	22/50 44.0%	0/11 0%	1.25	0.005	3/8 37.5%	0/9 0%	1.10	0.082

**Fig. 1:**
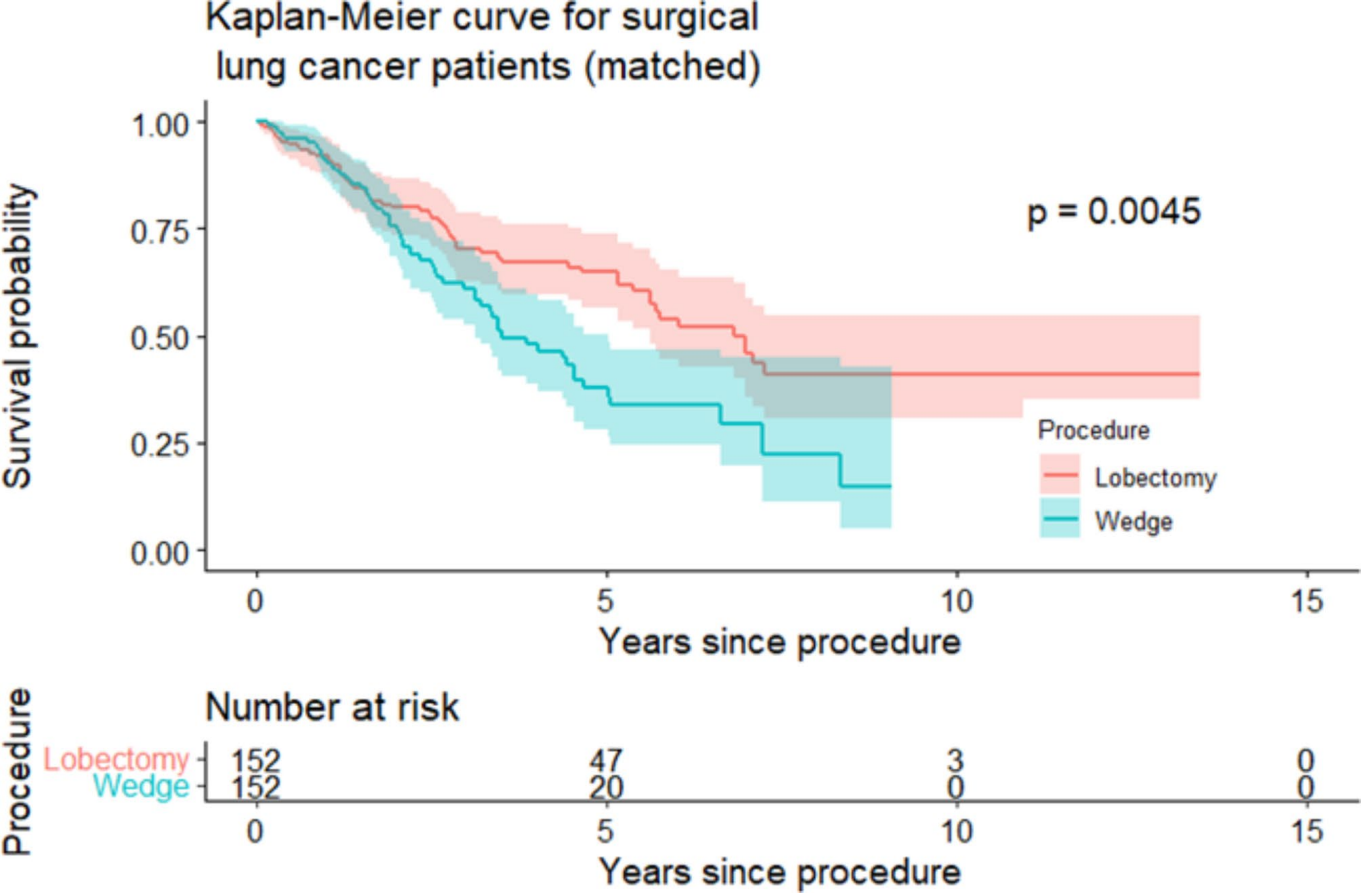
Kaplan-Meier curve for surgical lung cancer patients (matched)

The primary outcome for the study was overall survival. Secondary outcomes were morbidity, mortality, and hospital length of stay. To explore survival rates across the two surgical groups in the matched set, Kaplan-Meier estimates of survival were calculated and plotted (Fig. 1). Differences between groups were tested using a log-rank test, deemed significant with p < 0.05. Using a matched set means that any observed difference is theoretically more attributable to the choice of procedure. Characteristics of this matched set are presented in Table 1. The difference in survival is also presented as a Restricted Mean Survival Time (RMST), defined as the average time survived within a restricted/fixed post-operative time period. This allows a concrete interpretation and comparison of the mean survival of patients in each surgical procedure group in the five-year post-operative period.

## Results

Pre- and post-operative characteristics of the full data set (n = 618), categorised into lobectomy and wedge resection groups, are shown in Table 1. A total of 187 patients underwent wedge resection and 431 underwent lobectomy. Most of the post-operative histological findings were non-small cell lung cancer (NSCLC) (91%). Median hospital length of stay was 7 days for lobectomy and 5 days for wedge resection. In the overall cohort 5-year survival for the wedge group was 36.2% versus 64.3% for lobectomy (SMD 0.58, p < 0.001). Statistically significant differences in patient characteristics were that lobectomy patients tended to be younger, had a lower prevalence of cardiac morbidity and had better performance status and pre-operative lung function.

### Cox modelling

Cox models were fitted to all 618 patients with T1 stage tumours. The final model adjusted for surgical group included the following variables: age (over 70 years), sex, smoking status, predicted forced ejection volume (FEV1), performance ECOG, ASA grade and N-stage (N0 or not).

Testing of the proportional hazards assumption showed borderline violation for the surgical group covariate (p = 0.08); visual inspection of Kaplan-Meier curves (not shown) also suggested that the effect of group was negligible before 1.5-2 years when the curves clearly diverged. A time-varying coefficient was introduced that allowed the effect of surgical group to be different before and after this threshold. Thresholds from 1 to 3 years in steps of 0.1 year were tested and the model found to have lowest AIC at around 1.6 years. The Cox model with time-varying coefficient of group satisfies the proportional hazards assumption (p = 0.75) with lowest AIC and improved concordance (0.67).

This model (Table 2) shows that the effect of surgical group is negligible before 1.6 years (HR 1.26, CI 0.75–2.11, p = 0.39) but that wedge resection carries a 3-fold risk of death beyond 1.6 years (HR 3.14, CI 1.98–4.99, p < 0.001), when controlling for other covariates.

**Table 2:** Cox proportional hazards model fitted to all t1 tumour patients (n = 618)

Covariate		Hazard ratio (Confidence Interval)	p-value
Age	*Reference: 70 or under*		
	Over 70	1.01 (0.73, 1.39)	0.97
Procedure (with time-varying coefficient)	*Reference: lobectomy*		
	Wedge, < 1.6 years post-op	1.26 (0.75, 2.11)	0.39
	Wedge, ≥ 1.6 years post-op	3.14 (1.98, 4.99)	**< 0.001**
Sex	*Reference: female*		
	Male	1.64 (1.19, 2.25)	**0.002**
N stage	*Reference: N0*		
	N1/N2/NX	1.86 (1.30, 2.66)	**< 0.001**
Smoking	*Reference: Current/Ex smoker*		
	Never smoked	0.77 (0.40, 1.50)	0.45
Performance ECOG	*Reference: Fully active*		
	Light work only	0.94 (0.64, 1.39)	0.75
	Mobile > 50%	0.55 (0.29, 1.04)	0.067
	Limited/immobile	1.86 (0.74, 4.68)	0.19
ASA grade	*Reference: Normal/healthy*		
	Mild systemic disease	1.18 (0.80, 1.74)	0.39
	Severe or life threatening	2.08 (1.28, 3.37)	**0.003**
FEV1 predicted		1.00 (0.99, 1.01)	0.77

### Propensity matching

Propensity matching resulted in a matched dataset of 304 patients whose characteristics are also described in Table 1. Pre-operative characteristics of patients were similar across surgical groups among variables not used for matching as well as those explicitly employed to match. Hospital length of stay was slightly longer for the lobectomy group (median 7 versus 5.5 days). 5-year survival was 66.2% as opposed to 38.5% (SMD 0.58, p = 0.003) for the wedge resection group.

### Restricted mean survival time

Considering the first five years post-operatively, we find that the difference in restricted mean survival is 0.49 years greater for patients undergoing lobectomy (lobectomy 3.91 years mean survival out of the first five post-operative years compared to wedge RMST 3.42 years, p = 0.016) in the matched set.

## Discussion

The only randomised control trial (RCT) comparing wedge resection versus lobectomy for primary lung cancer was carried out over 25 years ago and demonstrated worse survival after wedge resection [3]. Most evidence on this topic lies in retrospective and prospective cohort studies. A number of these have demonstrated at least equivalent 5 year survival rates [5], [17], whilst others have found the opposite [18]. Significant heterogeneity between studies makes it difficult to perform meta-analyses [19]. Despite this, a recent meta-analysis and systematic review which only included papers since the year 2000 found equivalence although it alluded that there have been no recent RCTs which it could include in the review [20].

The surgical approach has changed from open thoracotomy to VATS since the original RCT [3] was published. VATS has been demonstrated to improve short term perioperative morbidity and mortality. No inferiority to lobectomy was shown from an oncological point of view [21] and is therefore recommended by The European Society for Medical Oncology (ESMO) committee for all stage I lung cancers [10]. It would be sensible to hypothesise that wedge resection would be beneficial to patients in terms of parenchymal sparing, although this has been refuted, as compensatory lung growth may occur after lobectomy [22]. With the technological advancement of clinical imaging, surgeons may be more confident in their ability to remove a lesion with a lesser resection although this has its own inherent risks in terms of increased recurrence rate [23, 24]. Few reports claimed that surgical quality of a wedge resection with negative margins along with the examination of > 5 lymph nodes confers a survival advantage over a poor quality resection treated with additional radiotherapy [25]. Lymph node sampling during wedge resection is associated with better survival if for no other reason than to guide future treatment [16]. One study determined that wedge, segmentectomy and lobectomy are comparable oncologic procedures for patients with tumour size of 1 cm or smaller [26]. Due to this, segmentectomy has gained traction in Europe, but its uptake in the UK seems to be slower. Segmentectomy as opposed to wedge resection is demonstrated to be more superior in terms of overall survival, likely because of lymph node dissection leading to a reduction in recurrence and metastases [27].

Another study revealed for patients over 71 years of age, 5 year survival rates of wedge resection may be equivalent to that of lobectomy [28]. In our study we have seen survival superiority for lobectomy over wedge resection in the matched patients. The reasons lobectomy has better long survival rates is probably due to the wide surgical margin and better lymph node dissection [25].

Tumour size over staging is used to determine resection type. This poses a conundrum in terms of data collection and whether the tumour, node, metastases (TNM) staging system [15] is appropriate for the staging of lung cancer or whether it should be further differentiated. Biologic characteristics and histology of the malignancy also affect survival so patients cannot be treated as a ‘one size fits all’ approach. With the advent of targeted lung health checks in the UK, it is likely that an increasing number of smaller nodules will be found which is why we need to determine the best choice of treatment for these patients. We minimised selection bias following propensity matching; after adjustment for age and pre-op characteristics, and even in a balanced set of early-stage disease patients, lobectomy patients still have superior survival to wedge resection patients at 5 years.

### Limitations

Several limitations are acknowledged with regards to our study. As this is a retrospective study, data was collected over a significant time frame. Some data was missing. 618 patients were identified to have T1 stage tumours from the initial data. Pre-operative T-stage was used to split off the groups for propensity matching, but the post-operative classification did not always match this evaluation. The choice of treatment was made when only the pre-operative information was available and that is the variable under discussion here – what effect did the decision about treatment have on the outcome. Only a proper randomised trial, where treatment decision is not affected by the clinical characteristics of patient/tumour, can avoid the indication bias. Treatment really is almost pre-determined by covariates which makes it difficult to untangle from outcomes. Improvements in diagnostics and treatment may also have an impact on the data.

## Conclusion

In our study, lobectomy is associated with superior overall survival compared to wedge resection in patients with T1 tumours and should be the standard of care for all patients who are fit enough to undergo surgical resection. In the short-term, the effect of procedure is negligible. However, after around 1.6 years, lobectomy is associated with improved survival. Clinicians should be cautious in whom they offer wedge resections to due to the possible negative impact upon long term survival especially in the context of other available treatment options.

## Data Availability

Not applicable.

## Abbreviations

AIC: Akaike Information Criterion
ASA Grade: American Society of Anesthesiologists physical status grade
BMI: Body Mass Index
BTS: British Thoracic Society
ECOG: Eastern Cooperative Oncology Group Performance Status
ESMO: The European Society for Medical Oncology
FEV1: Forced expiratory volume in 1 s
FVC: Forced Vital Capacity
NHS: National Health Service
PATS: Patient Advocate Tracking System
PDS: Personal Demographics Service
NSCLC: Non-small cell lung cancer
RCT: Randomised control trial
SCTS: Society for Cardiothoracic Surgery in Great Britain and Ireland
SMD: Standardised Mean Difference
T_1_: Tumour stage 1
TNM: Tumour, Node, Metastases
VATS: Video assisted thoracoscopy
